# A hTfR1 Receptor-Specific VHH Antibody Neutralizes Pseudoviruses Expressing Glycoproteins from Junín and Machupo Viruses

**DOI:** 10.3390/v16121951

**Published:** 2024-12-20

**Authors:** Qinglin Kang, Gege Li, Yan Wu, Shaoyan Wang, Zhengshan Chen, Xiaodong Zai, Xiaoyan Pan, Rong Wang, Jiansheng Lu, Peng Du, Zhixin Yang, Xiangyang Chi, Gengfu Xiao, Junjie Xu

**Affiliations:** 1 School of Medicine, Zhejiang University, Hangzhou 310063, China; kql_lynn@163.com; 2 Laboratory of Advanced Biotechnology, Beijing Institute of Biotechnology, Beijing 100071, China; 15382129909@163.com (G.L.); luckywsyhhh@126.com (S.W.); czs0076@163.com (Z.C.); zaixiaodong@163.com (X.Z.); wangrong_8312@163.com (R.W.); lujiansheng2008@163.com (J.L.); dudedu@sina.com (P.D.); yy_xiao@126.com (Z.Y.); xiangyangchi@163.com (X.C.); 3 State Key Laboratory of Virology, Wuhan Institute of Virology, Chinese Academy of Sciences, Wuhan 430071, China; wuyan@wh.iov.cn (Y.W.); panxy@wh.iov.cn (X.P.); xiaogf@wh.iov.cn (G.X.)

**Keywords:** Junín viruses, Machupo viruses, VHH antibody, transferrin receptor 1

## Abstract

The Junín virus (JUNV) is one of the New World arenaviruses that cause severe hemorrhagic fever. Human transferrin receptor 1 (hTfR1) has been identified as the main receptor for JUNV for virus entry into host cells. To date, no treatment has been approved for JUNV. Herein, we investigated 12 anti-hTfR1 VHH (variable domain of the heavy chain of heavy-chain antibody) antibodies and confirmed their interaction with hTfR1. Most of them could bind to the hTfR1 apical domain, which is the glycoprotein 1 (GP1) binding domain of JUNV. Among them, 18N18 exhibited neutralizing activity against both the human immunodeficiency virus (HIV)-vectored lentiviral Junín pseudoviruses and the recombinant vesicular stomatitis virus (VSV)-vectored Junín pseudoviruses. We also verified that 18N18 blocked the interaction between hTfR1 and JUNV GP1. In addition, 18N18 could neutralize another New World arenavirus, the Machupo virus. Using AlphaFold 3-based simulation of 18N18–hTfR1 docking, we determined that 18N18’s binding epitope was located at the JUNV GP1 binding epitope. 18N18 represents a candidate for JUNV treatment and provides a potential approach that could be applied to New World arenaviruses.

## 1. Introduction

Several New World arenaviruses cause human hemorrhagic fevers. Among them, Argentine hemorrhagic fever, caused by Junín virus (JUNV) infection, and Bolivian hemorrhagic fever, triggered by Machupo virus (MACV) infection, are two severe hemorrhagic fever diseases that are mainly prevalent in South America [1]. Both exhibit high mortality rates of about 15–30%, thus posing a significant public health threat [2]. These diseases are mainly transmitted to humans via rodents. The clinical features include fever, severe myalgia, bleeding tendency, shock, nerve abnormalities, leukopenia, and thrombocytopenia. The rodents, the drylands vesper mouse (*Calomys musculinus*) and the large vesper mouse (*Calomys callosus*), are the primary natural hosts of JUNV and MACV, respectively [2,3]. For rodents, infection is usually mild or asymptomatic. However, humans can be infected through contact with the rodents or their secretions, excretions, contaminated food, or virus-carrying aerosols, subsequently developing hemorrhagic fever.

To date, research on MACV has been limited, and there are no approved vaccines or specific therapeutics for MACV. For JUNV, there are only a few vaccines and antibodies in development. Candid#1, a live attenuated vaccine for JUNV, is only applied in the adult population at high risk in epidemic zones. Utilization of Candid#1 has been associated with a significant reduction in the incidence of AHF. Candid#1 was attenuated using a pathogenic JUNV isolate (XJ), serially passed through guinea pigs and mice in cell culture [4]. However, there is a greater safety risk associated with administering live attenuated vaccines to children, pregnant women, and individuals with weakened immune systems [5,6]. Moreover, there are no specific antiviral medicines available for the clinical treatment of JUNV infections. Ribavirin and favipiravir, categorized as broad-spectrum antiviral drugs, have exhibited some activity against the JUNV and have been utilized in specific clinical scenarios [7,8]. However, these drugs are only moderately effective in primate models [9,10,11].

The only standard specific treatment for this disease is the transfusion of convalescent plasma [1]. Researchers have demonstrated that antibodies titers in immune plasma correlate directly with the therapeutic efficacy achieved [12]. Nevertheless, treatment using this method is effective only during the first week of infection [12,13,14]. Furthermore, plasma therapy has many drawbacks, such as some advanced neurological syndromes in patients treated with plasma, the limited availability of immune plasma, and the risk of transfusion-transmitted diseases [1]. Although experts suggest using Candid#1 immune plasma as an alternative, the neutralizing antibody titers in vaccinated individuals are significantly lower compared with those in convalescent patients [15].

To circumvent the above risk, antibody-based therapies play a crucial role in countering viral infections. Various targets are under investigation to generate antibodies against JUNV [16,17,18]. Neutralizing antibodies targeting the JUNV glycoprotein precursor (GPC) are among the most extensively studied. Similarly to other arenaviruses, envelope GPC forms the spike on the surface of the virus, which is essential for viral adhesion to host cells and membrane fusion. GPC consists of stable signaling peptides (SSPs), envelope glycoprotein-1 (GP1), and envelope glycoprotein-2 (GP2). The head of the glycoprotein spikes is made up of a GP1 trimer [19]. Therefore, relevant antibodies typically prevent the virus from binding to its receptor by targeting GP1. GD01 and OD01 [20], two of the earliest JUNV-neutralizing antibodies, have been subjected to detailed structural studies, making substantial contributions to the body of knowledge regarding JUNV neutralization [21,22]. A humanized antibody, hu99, has shown some protective efficiency in non-human primates [18]. In another study, various neutralizing antibodies were isolated from an individual vaccinated with Candid#1, and their binding epitopes and broad spectrum were analyzed [23]. In that study, CR1-28 was identified as a neutralizing antibody against JUNV with a half-maximal inhibitory concentration (IC_50_) of 0.09 μg/mL. CR1-07 is another neutralizing antibody, which has an IC_50_ of 0.63 μg/mL. In addition, some antibody candidates targeting GP1, such as JUNV1 [24] and J99 [25], have also been verified in pseudoviruses or in live attenuated vaccine Candid#1 to have certain neutralizing activities. Antibodies targeting the viral N protein have also been screened to neutralize Candid#1; however, they failed to block the pathogenic JUNV strain [17]. Although there are already some JUNV-neutralizing antibodies, they have a limited efficacy against MACV and other New World arenaviruses. In particular, antibodies that target GPC have difficulty achieving broad-spectrum neutralization, possibly because of substantial sequence variability at the receptor binding site. The GP1 segment exhibited a sequence identity that varied between 24% and 44% in JUNV, MACV, and certain other New World arenaviruses [23]. Specifically, MACV has a longer loop 10, which is different from JUNV and other New World arenaviruses, and this poses a barrier to antibody cross-neutralization. This has been analyzed in studies of OD01, GD01, and CR1-28 [23,26].

There is evidence that the human transferrin receptor 1 (hTfR1) acts as the primary infectious receptor for both JUNV and MACV. The expression of hTfR1 in hamster cell lines substantially enhanced infection by JUNV and MACV pseudoviruses [27]. Some properties of hTfR1 suggest a potential role in the replication and pathogenesis of arenaviruses. For instance, hTfR1 can be rapidly endocytosed into an acidic vesicle, which is consistent with the requirement for an acidic environment for viral infection [28]. hTfR is expressed at high levels on rapidly dividing cells, including macrophages and activated lymphocytes, which are also prime targets for arenavirus infection [15,27]. In addition, hTfR1 is highly expressed in endothelial cells, which are considered pivotal in the pathogenesis of viral hemorrhagic fevers [29]. Moreover, the upregulation of hTfR1 on immune cells activated by the infection could potentially enhance viral replication within these cells, which accounts for the observed high lethality [27]. Structural analysis of hTfR1 and virus GP1 confirmed that the viruses mainly bind to the apical domain of hTfR1 [30]. Moreover, JUNV and MACV bind to almost the same epitope of hTfR1 [30]. Therefore, antibodies targeting the receptor, hTfR1, might have a broad ability against New World arenaviruses. There are also antibodies targeting hTfR1 that are used to block JUNV infection; for example, Ch128.1 effectively inhibits the invasion of various New World hemorrhagic fever arenaviruses [16,31].

Most previously studied antibodies are traditional IgG antibodies and no anti-hTfR1 VHH antibody (variable domain of the heavy chain of heavy-chain antibody) has been developed for JUNV infection. A single-domain antibody (sdAb) is a fragment only containing the variable region of heavy-chain antibodies, including the VHH from camels or alpacas and the VNAR (variable domain of new antigen receptor) from rays or sharks [32]. These sdAbs are only nano-sized; therefore, they are easier to engineer or construct with other drugs or antibodies [33]. SdAbs have a long complementary-determining region 3 (CDR3), which provides access to cryptic epitopes that conventional monoclonal antibodies cannot reach [33]. Furthermore, their good water solubility is conducive to large-scale production and formulation development in the process of drug research and development [33,34,35].

Herein, we describe a chimeric VHH antibody, 18N18, which was isolated from an alpaca immune library using phage display and fused with human Fc. 18N18 exhibited high affinity for hTfR1 and specifically bound to the apical domain. We tested the neutralization ability of 18N18 against both the human immunodeficiency virus (HIV)-vectored lentiviral Junín pseudoviruses and the recombinant vesicular stomatitis virus (VSV)-vectored Junín pseudoviruses. Then, using bio-layer interferometry (BLI) technology, we confirmed that 18N18 could block the interaction between JUNV GP1 and hTfR1. The neutralizing activity of 18N18 against HIV-vectored Machupo pseudoviruses, another New World arenavirus, was also assessed. The result indicated that 18N18 could also neutralize pseudotyped MACV. Subsequently, we simulated its interaction with hTfR1 computationally and confirmed the competitive inhibition between the antibody and JUNV GP1. In summary, 18N18 is a potential candidate molecule for JUNV- and MACV-neutralizing antibodies that target the receptor, providing a reference for future research into New World arenaviruses.

## 2. Materials and Methods

### 2.1. Immunization of the Alpaca

The alpaca (*Lama pacos*) was immunized with hTfR1. Briefly, purified hTfR1 was mixed with Ferund’s complete adjuvant for the first immunization or with incomplete adjuvant for the following immunizations. After full emulsification, the mixture was injected into a healthy adult alpaca at multiple points, with an interval of 14 days. Alpaca serum was collected after the fourth and fifth immunizations. The serum antibody against hTfR1 titer was measured using an enzyme-linked immunosorbent assay (ELISA) with pre-immunization serum used as a negative control. The secondary antibody for titer detection was horseradish peroxidase (HRP)–Goat anti-Llama IgG H&L (Catalog No. ab112786, Abcam, Cambridge, UK) for IgG or HRP–Rabbit anti-Camelid VHH (Catalog No. A01861, Genscript, Nanjing, China) for VHH. After determining that a high antibody titer had been obtained, a booster immunization was performed.

### 2.2. Construction of the Phage Display VHH Library

The method of constructing VHH phage display library was based on that detailed in a previous study [36]. Seven days after the booster immunization, whole blood was collected, and lymphocytes were separated using a lymphocyte isolation solution, before extracting and reverse transcribing the total RNA. After amplification of the VHH-coding gene using two rounds of PCR, the fragment and the vector (pMECS) were digested with Pst I and Not I enzymes, and ligated together using T4 DNA ligase. The ligation products were electrotransformed into receptive *Escherichia coli* SS320 cells. The electrotransformed SS320 cells were diluted in a 10-fold gradient and plated on selective medium. The number of clones was calculated at an appropriate dilution level. Clones were picked, and their DNA was extracted and sequenced to determine the insertion of the VHH gene into the phage vector, the integrity of the gene, and the accuracy of the reading frame. The correct insertion rate and the diversity of the library were evaluated based on the sequencing results.

### 2.3. Panning of the hTfR1-Specific Phage Display VHH Library

The hTfR1 protein (Catalog No. 11020-H07H, Sino Biological, Beijing, China) was coated on an immunotube at 4 °C overnight for panning of specific phage clones. It was used at 10 µg/mL in the first round of coating and at 1 µg/mL in the second round. After coating, the immunotube was blocked with blocking solution (phosphate-buffered saline [PBS] containing 2.5% skim milk powder) and incubated at room temperature for 2 h. Subsequently, the VHH library was added to the blocked immunotube and incubated at room temperature for 2 h. After 10 washes with PBS-Tween20 (PBST) and 5 washes with PBS, the bound phage was eluted using 1 mL of elution buffer (0.1 M Glycine–HCl, pH 2.2), and then neutralized with neutralization solution (1 M Tris–HCl, pH 8.0). Next, *E. coli* XL1-Blue grown to the logarithmic phase was infected with the eluted phage, incubated at 37 °C for 30 min with rotation at 150 rpm, and centrifuged at room temperature (2000× *g*, 10 min). The pellet was suspended in fresh medium and cultured on plates. After two rounds of panning, single colonies were prepared and used for further identification.

### 2.4. Screening of hTfR1-Specific Positive Clones

The hTfR1 protein was coated on a 96-well microplate (9018, Costar, Washington, DC, USA). An irrelevant antigen with a His-tag and bovine serum albumin (BSA) were coated as negative controls. After blocking with blocking solution, the single-phage colonies were added and incubated at 37 °C for 1 h. The wells were washed 12 times with PBST and then treated with 2 µg/mL HRP-labeled M13 monoclonal antibodies (Catalog No. 1973-MM05T-H, Sino Biological, Beijing, China) and incubated at 37 °C for 45 min. After washing with PBST, tetramethylbenzidine (TMB; Catalog No. E-IR-R201, Elabscience, Wuhan, China) was used to detect the positive clones. The reaction was stopped by adding 2 M H_2_SO_4_ (50 µL per well), and the absorbance was measured at 450 nm and 595 nm using a microplate reader (Molecular Devices, Sunnyvale, CA, USA).

### 2.5. Cloning, Expression, and Purification of VHH-Fc

The positive clones were used as templates to amplify the VHH-encoding fragments. The amplified fragments were then cloned separately into the pABG-Fc expression vector. The constructed plasmids encoded VHH-hFc fusion proteins as chimeric heavy-chain antibodies. The recombinant VHH-hFc was expressed using Free-Style™ HEK293-F cells (Thermo Fisher Scientific, Waltham, MA, USA) and then purified using a HiTrap MabSelect Xtra purification column (Cytiva, Uppsala, Sweden) in an AKTA Pure purification system (GE Healthcare, Piscataway, NJ, USA). The molecular weight and purity of the chimeric heavy-chain antibodies were confirmed using sodium dodecyl sulfate–polyacrylamide gel electrophoresis (SDS-PAGE).

### 2.6. Enzyme-Linked Immunosorbent Assay

The binding ability of candidates to hTfR1: A 96-well microplate (9018, Costar, Washington, DC, USA) was coated with 100 ng/well of hTfR1 (Catalog No. 11020-H07H, Sino Biological, Beijing, China) and incubated at 4 °C overnight. Following incubation, the plate was blocked with 2.5% skim milk. The candidate VHH-hFcs were diluted starting at a concentration of 1 µg/mL, followed by a 3-fold gradient dilution, and then added to the 96-well plates and incubated at 37 °C for 1 h. Subsequently, the plates were washed with PBST and then incubated with HRP-labeled goat anti-human IgG (Catalog No. ZB-2304, ZSGB-BIO, Beijing, China) at 37 °C for 45 min. Finally, the plates were washed using PBST, and TMB was added to develop the color. The reaction was stopped using 2 M H_2_SO_4_ (50 µL/well) and the absorbance was measured at 492 nm and 630 nm using the microplate reader. The binding ability of the antibodies to hTfR1 was determined as the 50% effective concentration of the antibodies (EC_50_).

The binding ability of candidates to the hTfR1 apical domain: We expressed the hTfR1 apical domain (D184-E383) in FreeStyle^TM^ HEK293-F cells. The binding ability detection of candidates to the hTfR1 apical domain was performed according to the method used for the binding detection of candidates to hTfR1, with antibody concentrations adjusted to 10, 1, and 0.1 μg/mL.

### 2.7. KD Analysis by the Biacore System

The Biacore™ T200 System (Cytiva, Shanghai, China) was used to determine the dissociation constant (KD) of VHH-hFc, based on surface plasmon resonance (SPR). The purified candidates were diluted to 25 nM using Hepes Buffered Saline (HBS)–EP+ buffer (Catalog No. BR100669, Cytiva) and fixed to the protein A sensor (Catalog No. 29127555, Cytiva). Then, hTfR1 was diluted in an appropriate gradient and was associated with the sensors. Finally, the dissociation steps were performed. After detection, the data were analyzed using the Biacore™ T200 Evaluation Software 3.2 to calculate the KD value.

### 2.8. Thermostability Assessment

The candidates’ thermostability was assessed using Uncle (UNchained Laboratories, Pleasanton, CA, USA) based on fluorescence, dynamic light scattering (DLS), and static light scattering (SLS). The sample was diluted in PBS to 1.5 µg/mL and the temperature was increased from 25 °C to 95 °C at 0.25 °C/min. The barycentric mean (BCM) of the fluorescence intensity was measured to determine the melting temperature (Tm) of the antibody. The SLS data were also gathered at 266 nm to analyze the aggregate temperature (Tagg). Before heating, the particle size and polydispersity were measured using DLS.

### 2.9. Western Blotting

Protein samples were prepared and separated using SDS-PAGE, followed by electrotransfer onto methanol-activated polyvinylidene fluoride membranes. Then, the membranes were blocked using a sealing solution (1 × Tris-buffered saline (TBS) + 5% skim milk powder) at room temperature for 1 h with rotation at 60 rpm. Then, the detection antibody, HRP-Conjugated His-Tag Monoclonal Antibody (Catalog No. HRP-66005, Proteintech, Wuhan, China), was diluted with sealing solution to 1:5000, added to the membrane, and shaken at 60 rpm at room temperature for 1 h. The membranes were then rinsed six times using TBST (1 × TBS + 0.1% Tween-20). Finally, the immunoreactive proteins on the membrane were visualized using a chemiluminescent substrate (RPN2106, GE Healthcare-Amersham, Amersham, UK).

### 2.10. Preparation of Anti-TfR1 Antibody Ch128.1

Ch128.1 is an anti-hTfR1 IgG antibody which has been proved to neutralize infection with broad-spectrum New World arenaviruses [31]. In this study, ch128.1 was used as a control. The sequences of ch128.1 were derived from patent (US6329508) and crystal structure (PDB: 6WLA) documents, and fused with human IgG Fc. The expression and purification were the same as expression and purification of VHH-Fc.

### 2.11. Neutralization Assay Against HIV-Vectored Lentiviral Junín and Machupo Pseudoviruses

Firstly, we constructed JUNV or MACV pseudoviruses. The GPC gene sequence of the JUNV MC2 strain (Taxon ID: 2169991) or MACV Carvallo (Taxon ID: 3052317) was codon-optimized for expression in human cells and amplified in the pDC316 vector as the envelope plasmid. The plasmid was then transfected into HEK293T cells together with the lentiviral plasmid, pNL4-3.Luc.R-E- (Catalog No. 3418, NIH AIDS reagent program, Germantown, MD, USA). After 2 to 3 days of culture, the supernatant was collected to obtain the pseudoviruses. The titer of the pseudoviruses in HEK293-hTfR1 cells (GM-C30180, Genomeditech, Shanghai, China) was measured using a Bright-Lite Luciferase Assay System (Catalog No. DD1204-02, Vazyme, Nanjing, China). For the neutralization assay, the antibody candidates were diluted and mixed with the HEK293-hTfR1 cells. After incubation at 37 °C for 2 h, the pseudovirus (diluted to 1 × 10^5^ RLU) was added, and then the luciferase activities were measured after 48 h of culture at 37 °C. The percent neutralization (inhibition rate) was calculated as 100% − (sample signals − blank control signals)/(virus control signals − blank control signals) × 100%. The neutralizing ability of the antibodies was determined as the 50% inhibitory concentration of the antibodies (IC_50_).

### 2.12. Neutralization Assay Against VSV-Vectored Junín Pseudoviruses

The packaging of recombinant VSV (vesicular stomatitis virus) expressing JUNV GP (rVSVΔG-eGFP/JUNV GPC) has been described previously [37]. Here, we analyzed the neutralization of candidates against rVSVΔG-eGFP/JUNV GPC. First, HEK293-hTfR1 cells were seeded in 24-well plates. When the cell density reached 90%, the antibody was diluted in a 4-fold gradient starting at the initial concentration of 5 μM and added to the well plate. After incubation at 37 °C for 1 h, the virus was diluted to 1000 PFU/mL and added to 24-well plates, and then incubated at 37 °C for 1 h. The virus was then discarded, the cells were washed with PBS, and they were cultured in high-sugar DMEM medium containing 1% methylcellulose and 10% FBS (fetal bovine serum). After incubating at 37 °C for 24 h, the number of fluorescent plaques was counted under a fluorescence microscope. The inhibition rate and IC_50_ of the antibodies against rVSVΔG-eGFP/JUNV GPC were calculated using the same method as for HIV-vectored lentiviral pseudoviruses.

### 2.13. Detection of Blocking of JUNV-GP1–hTfR1 Interaction by Bio-Layer Interferometry

The ForteBIO^®^ Octet QKe System (Pall ForteBio Corporation, Fremont, CA, USA) was used to determine the blocking of the JUNV-GP1–hTfR1 interaction, based on BLI. First, hTfR1 (200 nM) in HBS-EP (Catalog No.AA7211, Kezhou, Beijing, China) was loaded onto nitrilotriacetic acid (NTA) sensors (Catalog No.18-5101, Sartorius, Goettingen, Germany). Then, the candidates (200 nM in HBS-RP) or buffers (HBS-EP) were associated with the sensors. Finally, another association step was executed to analyze the interaction between sensors and the samples containing 500 nM JUNV GP1-Fc (Catalog No. EVV25501, Antibody System Laboratories, Schiltigheim, France) in HBS-EP.

### 2.14. Statistical Analysis

All statistical analyses were performed using GraphPad Prism 9.0 software (version 9.0, GraphPad Inc., San Diego, CA, USA). Data for the binding ability (ELISA) and neutralization are presented as the mean ± standard deviation (SD).

## 3. Results

### 3.1. Construction and Panning of the Anti-hTfR1 VHH Library

**Figure 1:**
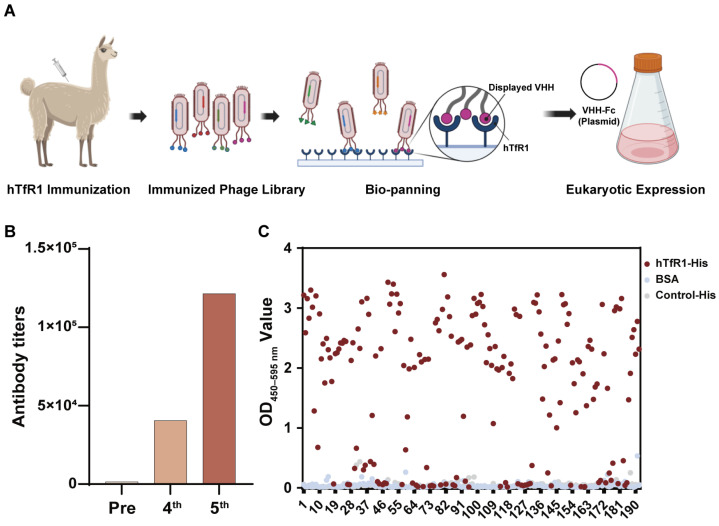
Construction and panning of the anti-human transferrin receptor 1 (hTfR1) nanobody library. (**A**) Schematic of phage-displayed VHH (variable domain of the heavy chain of heavy-chain antibody) antibody screening and expression. (**B**) Detection of anti-hTfR1 immunoglobulin G (IgG) titer in alpaca serum using an enzyme-linked immunosorbent assay (ELISA). The secondary antibody was horseradish peroxidase (HRP)–Goat anti-Llama IgG H&L antibody for specific IgG detection. (**C**) Monoclonal antibody identification from the second-round screening: The binding of phage-displayed monoclonal VHHs and hTfR1 was measured using ELISA with an HRP-labeled M13 monoclonal antibody. Bovine serum albumin (BSA) and an irrelevant protein with a His-tag served as two negative controls. The *X*-axis represents the number of monoclonal phage-displayed VHH antibodies. For a single VHH, its interactions with antigen hTfR1 and two controls are shown in different colors, in which a red dot represents its interaction with hTfR1 and a blue dot or a gray dot represents its interaction with BSA or control-His, respectively. OD—optical density.

The VHH screening program is shown in Figure 1A. We immunized the alpaca with hTfR1, and then the serum was collected before and after immunization to confirm the immunization efficiency. The ELISA results showed that the anti-hTfR1 antibody titers increased after immunization (Figure 1B) and the antibodies could specifically bind to hTfR1 (Figure S1A). After a booster immunization, alpaca peripheral blood mononuclear cells (PBMCs) were isolated to extract the RNA fragments encoding the VHH. The amplified VHH-encoding fragments were then cloned into the pMCES-gIII phage display vector and electrotransformed to construct a VHH library. The library capacity reached 3.2 × 10^8^ and the titer of the phage library was 1 × 10^13^ pfu/mL. After two rounds of panning, we sequenced the positive clones and obtained 12 different VHH sequences (Figure 1C). They were subsequently inserted into the expression vector pABG-hFc containing the constant region gene of the human Fc fragment to express the chimeric heavy-chain antibodies, VHH-hFc. These VHH antibodies were purified using the AKTA purification system. Their molecular weight was determined using SDS-PAGE as approximately 80 kDa under non-reducing conditions and 40 kDa under reducing conditions (Figure S2), which corresponded to the theoretical molecular weight of the chimeric heavy-chain antibody.

### 3.2. Basic Characteristics of hTfR1-Specific VHH Antibodies

**Figure 2:**
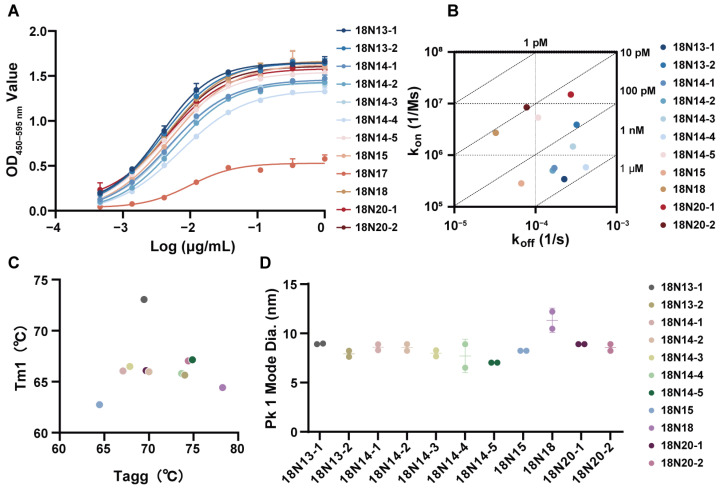
Basic characteristics of the candidate antibodies. (**A**) The binding ability of the candidates to hTfR1, as measured using ELISA. The initial concentrations of all candidates were 1 μg/mL, followed by a 3-fold serial dilution. Data are representative of at least two independent experiments. Means with the standard deviation (SD) are shown. (**B**) Affinity detection of the candidates binding to hTfR1 using surface plasmon resonance (SPR). (**C**) Thermostability of the candidate antibodies. Tm1 represents the temperature of the first thermal melting. (**D**) Protein size of the candidate antibodies: Pk1 Mode diameter represents the mode particle size of the first peak, which is also the main peak in the current test. The thermostability (**C**) and protein size (**D**) were measured using Uncle.

The binding ability of the 12 candidate antibodies with hTfR1 was determined using ELISA. Except for 18N17, all the candidates exhibited strong binding with hTfR1 (Figure 2A). The EC_50_ values were all less than 0.01 μg/mL (Table 1). Then, SPR was employed to further verify the affinities of other VHH-hFc molecules, revealing a strong binding with hTfR1 (Figure 2B and Figure S3; Table 1). All candidates exhibited a high affinity at the nanomolar level. Candidate 18N20-2 exhibited the highest affinity at 9.18 × 10^−12^ M. The affinities of 18N18, 18N20-1, 18N14-5, and 18N13-2 ranged from 1.2 × 10^−11^ M to 8.23 × 10^−11^ M. The affinities of the other candidates also reached at least 7.17 × 10^−10^ M.

We also evaluated the thermostability of all the candidates, which suggested that all of them were thermally stable (Figure 2C, Table S1). Taking 18N18 as an example, its melting temperature for the first transition (Tm1) was 64.42 °C, indicating that 18N18 had good thermal stability. According to the Tagg results, the aggregation temperature of 18N18 was 78.25 °C. Other candidates also showed a good performance. The kinetic diameter of the hydration of the candidates was then investigated using DLS. At 25 °C, the hydrodynamic diameters of all candidates were around 10 nm, which was consistent with the properties of chimeric heavy-chain antibodies (Figure 2D). Overall, the 12 candidates had good thermal stability, indicating their suitability for continued pharmacological development.

### 3.3. Binding of hTfR1-Specific Antibodies to the hTfR1 Apical Domain

**Figure 3:**
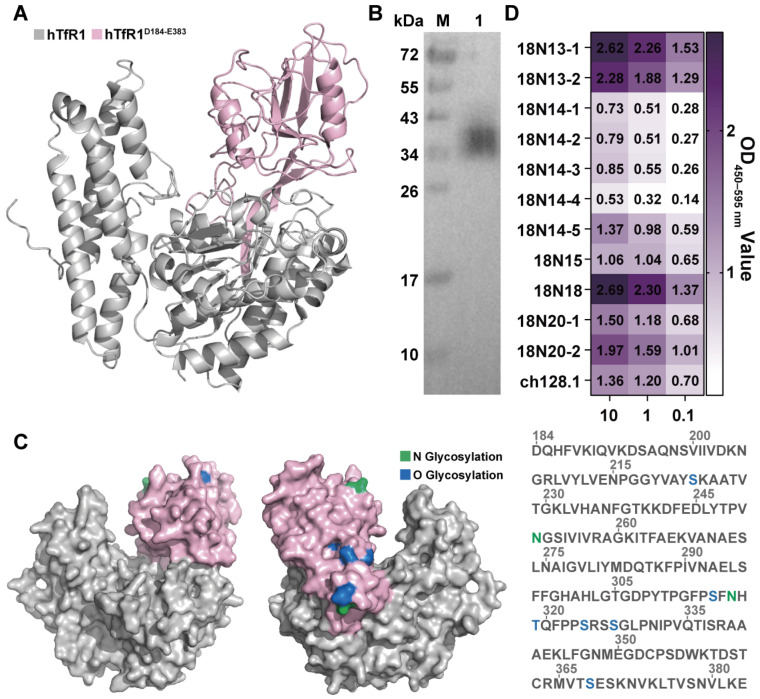
Binding of VHH antibodies to the hTfR1 apical domain. (**A**) The hTfR1 apical domain region is labeled in pink, hTfR1^D184-E383^. (**B**) Western blot detection of hTfR1^D184-E383^ expression. The detection antibody was the HRP-conjugated His-tag monoclonal antibody. M: Marker; Lane 1: purified hTfR1^D184-E383^. (**C**) Analysis of the glycosylation sites of hTfR1^D184-E383^. The N glycosylation sites are marked in green, and the O glycosylation sites are marked in blue. The numbers above the sequence represent the amino acid positions. (**D**) The binding ability of candidate antibodies to hTfR1^D184-E383^ was measured using ELISA. The expressed hTfR1^D184-E383^ was first coated on 96-well microplates, and then the binding activities of different antibodies with hTfR1^D184-E383^ were tested at three different concentrations of candidates (0.1, 1, and 10 μg/mL). An anti-TfR1 antibody, ch128.1, was added as the positive control.

Other researchers have proved that the apical domain of hTfR1 is the site where JUNV binds to infect host cells [16,38]. Consequently, if the antibodies could bind to the hTfR1 apical domain, they are more likely to function as neutralizing agents against viral infection. Therefore, the apical domain of hTfR1, D184-E383 (hTfR1^D184-E383^) (Figure 3A), was expressed. The expression of recombinant hTfR1^D184-E383^ was confirmed by Western blot analysis (Figure 3B). The theoretical size of hTfR1^D184-E383^ is 25.840 kDa; however, on the Western blot, the protein appeared to be around 35 kDa. Analysis of the sequences by DTU Health Tech (Kongens Lyngby, Denmark) revealed that hTfR1^D184-E383^ has several glycosylation sites, which might account for the difference between the theoretical and observed protein sizes (Figure 3C). To confirm the functional activity of the expressed hTfR1^D184-E383^, we measured the binding of ch128.1, which is specific to the apical domain of hTfR1, to the expressed hTfR1^D184-E383^. The results demonstrated that the ch128.1 antibody was capable of binding to hTfR1^D184-E383^, suggesting that the expressed protein is suitable for further detection (Figure S4). Subsequently, we investigated the binding of 11 candidate antibodies to hTfR1^D184-E383^ using ELISA. The results showed that most candidates could bind to hTfR1^D184-E383^, indicating that they have the potential to inhibit virus infection (Figure 3D). In particular, antibodies 18N18 and 18N13-1 exhibited the strongest binding ability, with the OD_450–595 nm_ values of 2.69 and 2.62 at 10 μg/mL, respectively. Moreover, 18N13-2, 18N20-2, 18N20-1, and 18N14-5 also showed good binding activity with TfR1. Therefore, in subsequent studies, we focused on the neutralizing activities of these antibodies.

### 3.4. The Neutralization Capacity of 18N18

**Figure 4:**
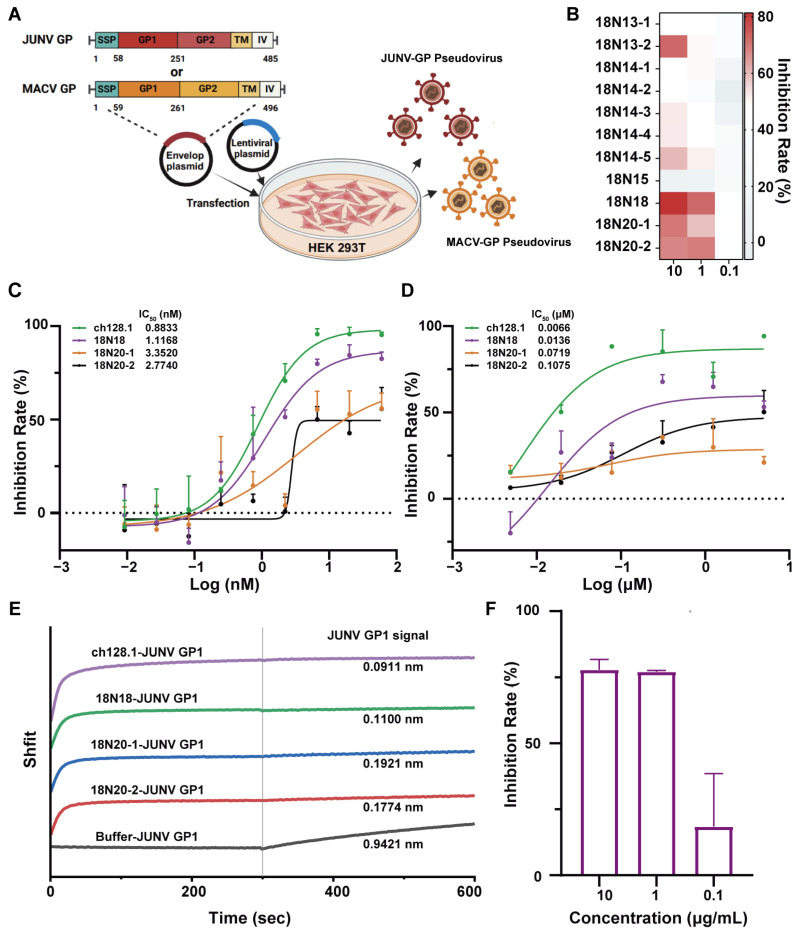
Screening of neutralizing antibodies against the Junín pseudovirus and the detection of the cross-neutralization against the Machupo pseudovirus. (**A**) Schematic diagram of HIV-vectored Junín pseudovirus construction. (**B**) Neutralization of JUNV by all candidates was measured at concentrations of 0.1, 1, and 10 μg/mL. (**C**) Neutralizing curves of 18N18, 18N20-1, and 18N20-2 against HIV-vectored Junín pseudoviruses. Ch128.1 was used as a positive control. The initial concentrations of all candidates were 60 nM, followed by a three-fold serial dilution. Data are representative of at least two independent experiments. Means with SDs are shown. (**D**) Neutralization of candidates against recombinant VSV (vesicular stomatitis virus) expressing JUNV GP (rVSVΔG-eGFP/JUNV GPC). The candidates start at 5 μM, followed by a four-fold serial dilution. (**E**) Detection of the blocking of hTfR1-JUNV GP1 interaction using Bio-Layer Interferometry (BLI). hTfR1 was first loaded onto nitrilotriacetic acid (NTA) sensors, and then allowed to interact with 18N18, ch128.1, or buffer. The interaction of GP1-Fc with the bio-sensor was finally measured. (**F**) Cross-neutralization ability against Machupo pseudoviruses of 18N18 at concentrations of 0.1, 1, and 10 μg/mL were measured using the same methods of neutralization against JUNV. Data are presented as means with SDs. IC_50_—half-maximal inhibitory concentration; GP1—glycoprotein 1.

There are biosafety concerns surrounding the use of the pathogenic Junín virus; therefore, HIV-vectored Junín pseudoviruses were first constructed to assess antibody neutralization, which permits the evaluation of antibody efficacy within a reduced-risk framework. The codon-optimized full-length JUNV GPC was inserted into the pDC316 vector. The pDC316-JUNV and pNL4-3-Luc vectors were transfected into HEK293T cells, and pseudoviruses were harvested from the culture supernatant (Figure 4A). All the antibodies were preliminarily screened using a neutralization assay. Their neutralization capacity was measured at the three concentrations of 0.1, 1, and 10 μg/mL, respectively. Subsequently, the neutralizing activities of the candidates against the HIV-vectored JUNV pseudovirus were determined. The results showed that most candidates reached an inhibition rate of around 50% at 1 μg/mL. Here, we found that the 18N18, 18N20-1, and 18N20-2 exhibited better neutralization effects than other candidates (Figure 4B). Then, we further analyzed the IC_50_ of 18N18, 18N20-1, and 18N20-2, with ch128.1 set as the positive control (Figure 4C). Among them, 18N18 exhibited a good IC_50_ of 1.1168 nM, which was almost the same as that of ch128.1. The IC_50_ values of 18N20-1 and 18N20-2 were 3.352 and 2.774 nM, respectively (Figure 4C). Therefore, 18N18 exhibited the best neutralization ability against HIV-vectored Junín pseudoviruses.

Moreover, for a more accurate evaluation of the candidates, we also tested their neutralization against rVSVΔG-eGFP/JUNV GPC, which are recombinant VSVs expressing JUNV GP [37]. The result is shown in Figure 4D; the neutralization of 18N18 (IC_50_ = 0.0136 μM) was slightly weaker than that of ch128.1 (IC_50_ = 0.0066 μM) but better than that of 18N20-1 (IC_50_ = 0.0719 μM) and 18N20-2 (IC_50_ = 0.1075 μM).

To analyze the neutralization mechanism, the blocking of the interaction between JUNV GP1 and hTfR1 by the candidate antibodies was evaluated. hTfR1 was initially loaded onto the NTA sensor, after which the sensors were allowed to interact with ch128.1, 18N18, 18N20-1, 18N20-2, or buffers. Following this, the interaction between GP1-Fc and the sensors was measured. The results showed that after binding with the candidates, the binding of GP1-Fc to hTfR1 was significantly inhibited (Figure 4E). Moreover, the neutralizing activity correlated positively with the extent of inhibition (Figure 4B–D). Among the candidates, 18N18, similar to ch128.1, exhibited the highest inhibition of the JUNV GP1–TfR1 interaction. In summary, we clearly demonstrated that the interaction between GP1-Fc and hTfR1 was inhibited after binding to 18N18.

Previous studies suggested that antibodies targeting the receptor hTfR1 could achieve broad-spectrum neutralization against New World arenaviruses [16]; therefore, we constructed HIV-vectored pseudoviruses for MACV, another member of the New World arenaviruses. Utilizing the same method as that used for JUNV pseudovirus construction, an MACV pseudovirus was obtained, whose titer, as measured using fluorescence intensity, reached approximately 1 × 10^5^. The results of neutralization assays showed that 18N18 could neutralize the infection of cells by the MACV pseudovirus (Figure 4F). Thus, 18N18 exhibits neutralizing efficacy against both pseudotyped JUNV and MACV.

### 3.5. AlphaFold 3 Analysis of the Binding Epitopes of 18N18

**Figure 5:**
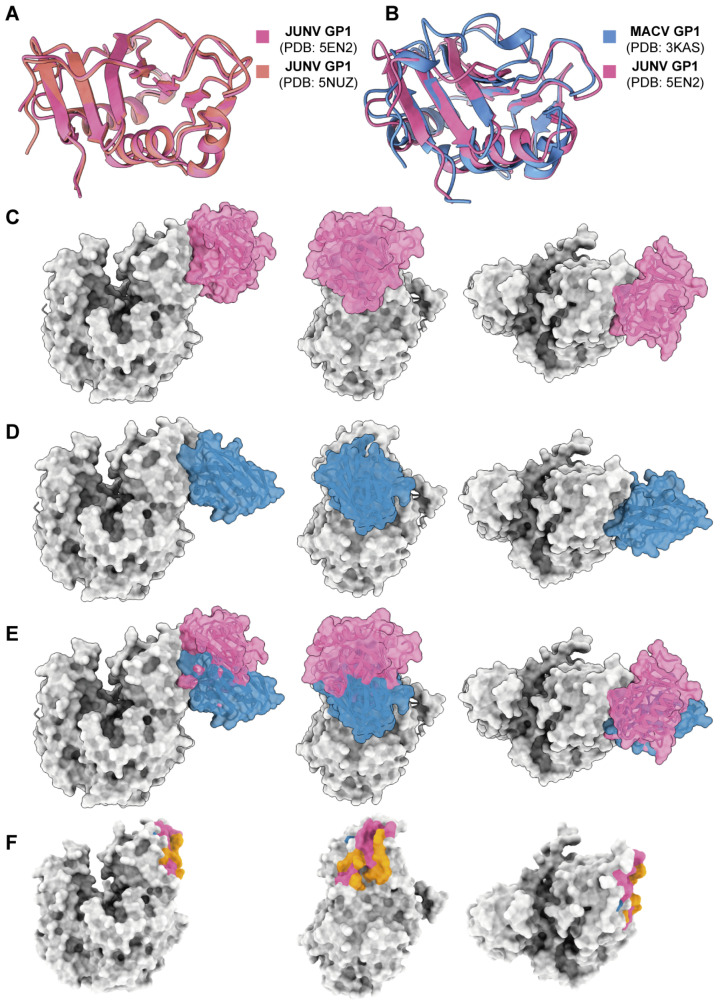
Epitope analysis of 18N18. (**A**) Alignment of two JUNV GP1 crystal structures, 5EN2 and 5NUZ. (**B**) Alignment of JUNV GP1 and MACV GP1 crystal structures, 5EN2 and 3KAS. (**C**) Simulation of the JUNV GP1-hTfR1 complex. (**D**) Prediction of the interaction between 18N18 and hTfR1 using AlphaFold 3. (**E**) Alignment of the JUNV GP1-hTfR1 complex and the 18N18-hTfR1 complex. (**F**) Epitope analysis of 18N18 and JUNV GP1 against hTfR1. For (**C**–**F**), the structure or epitope of 18N18 is shown in blue, and that for JUNV GP1 is shown in pink. The orange epitopes in F represent the overlap between 18N18 and JUNV GP1 binding epitopes.

Previous studies had solved the crystal structure of the MACV GP1–hTfR1 complex (PDB: 3KAS); however, there is no structure information on JUNV GP1–hTfR1. To analyze the mechanism by which 18N18 inhibits the interaction between JUNV GP1 and hTfR1, two JUNV GP1 crystal structures, 5EN2 and 5NUZ, were chosen. We compared their structures and found that they were basically the same (Figure 5A). The structure of JUNV GP1 (PDB: 5EN2) was selected as a representative and then compared with MACV GP1. The result showed high structural conservation between MACV and JUNV GP1 (Figure 5B). By structure alignment, superposition, and replacement, a conjectural structure of the JUNV GP1–hTfR1 complex was obtained (Figure 5C). AlphaFold 3 was then used to dock 18N18 and hTfR1, and the docking model with the highest ranking score was compared with the structure of JUNV GP1-hTfR1 (Figure 5D). Based on this model and evidence of competition from the BLI assay (Figure 4E), it was confirmed that 18N18 sterically hinders the interactions between JUNV GP1 and hTfR1 (Figure 5E,F). We also docked 18N13-1 and 18N15 to hTfR1 (Figure S5). The binding sites of these two antibodies to TfR1 were distinct from the binding sites of JUNV GP1, which was consistent with the results of neutralization experiment, and also explains the difference between binding activity and neutralizing activity.

## 4. Discussion

This study aimed to obtain an sdAb targeting hTfR1 that could neutralize JUNV. Through in vitro analysis, sdAb 18N18 exhibited neutralization ability against JUNV. In addition, we used BLI to confirm that the neutralizing effect of the sdAbs results from their blocking of the binding of Junín virus GP1 and hTfR1.

In previous studies, most antibodies targeted JUNV GP1, which is crucial for virus attachment. These neutralizing antibodies have different broad-spectrum neutralizing abilities due to their distinct binding epitopes. For instance, antibody CR1-07 could cross-neutralize MACV, whereas antibody CR1-28 could not. The reason for this difference is that, while the JUNV GP1 and MACV GP1 share a high degree of structural similarity, the distinctive loop 10 in MACV GP1 impedes the antibody binding of CR1-28. [23]. In addition, antibody epitopes targeting the virus might become inactivated if the virus undergoes mutations. Moreover, the high variability of GP1 among New World arenaviruses has also limited the development of broad-spectrum neutralizing antibodies.

Pathogenic New World arenaviruses bind to a shared region within the apical domain of hTfR1, where two major physiological ligands of hTfR1, transferrin (Tf) and hereditary hemochromatosis protein (HFE), do not bind [39,40,41]. Thus, developing antibodies targeting the apical domain of hTfR1 might be an effective therapeutic strategy to broadly inhibit infections by New World arenaviruses without substantially interfering with iron metabolism. Previous studies have confirmed that antibodies targeting hTfR1 can produce good neutralizing activity against JUNV [31]. In addition, because JUNV and MACV bind to similar epitopes of TfR1, antibodies that bind to those epitopes have the potential for broad-spectrum neutralizing activity, and this has also been verified in previous studies [16,30]. In this study, we confirmed that 18N18 can also neutralize the MACV pseudovirus.

Prior studies also indicated that anti-TfR1 therapy might impact iron uptake. Researchers have revealed that antibodies against TfR1 can produce certain cytotoxicity to cells [42]. Indeed, studies have reported that anemia appeared in monkeys after the administration of anti-TfR1 antibodies [42,43]. The antibody’s blocking of the interaction between Tf and TfR1, subsequently affecting iron uptake, was identified as a primary cause of this anemia. Additionally, some anti-hTfR1 antibodies have been reported to impact colony formation. The mouse anti-hTfR1 antibody V3/25, which does not inhibit Tf binding to hTfR1, the mouse IgG1 43/31, which inhibits the interaction between Tf and hTfR1, and the mouse anti-hTfR1 IgA 42/6, which inhibits Tf binding via steric hindrance, were studied [34,44]. The results of these studies suggested that the toxic effects of anti-hTfR1 antibodies depend partly on whether the antibody inhibits the binding of Tf to hTfR1. Moreover, Tf typically binds to the helical and protease-like domains of TfR1 [45]. Therefore, an antibody that binds to the apical domain of TfR1, which is farther from the Tf binding epitope, might be safer compared with antibodies that block Tf binding. Studies on antibody ch128.1 also confirmed that anti-hTfR1 antibodies targeting the hTfR1 apical domain appear to be safer than antibodies targeting regions that overlap Tf binding regions [31]. In our study, 18N18 targets the hTfR1 apical domain and does not interfere with the interaction between Tf and hTfR1; therefore, it might be safer than antibodies that block Tf. However, further experimental verification is needed.

18N18 is a VHH antibody, a type of antibody that exhibits several advantages in therapeutic development. They are easier to engineer because of their small size; therefore, they can be paired with other antibodies for enhanced neutralization, or conjugated with indicators to serve as research tools [46]. Moreover, VHHs are easily produced and highly soluble, which are good properties for medicinal use [32]. Indeed, several sdAbs have been approved for marketing, the first of which was Caplacizumab developed by Ablynx Inc., which is used to treat acquired thrombotic thrombocytopenic purpura. There are also neutralizing VHHs under evaluation in clinical trials, such as ALX-0171, also developed by Ablynx Inc., which could be used to treat respiratory syncytial virus infections.

However, sdAbs are small, with a molecular mass of around 15 kDa; therefore, they are eliminated more quickly than IgG because of the clearance of small molecules by the kidneys. This results in a shorter half-life, which ranges from a few minutes to tens of hours. Therefore, various approaches are usually employed to prolong their half-life. Fusion with an IgG Fc fragment is a common method, and herein, we used this method to prolong the half-life of the sdAbs for future in vivo evaluation and to make it easier to purify them. Other methods, such as fusion with human serum albumin (HSA) binding domains [47] or with anti-HSA antibodies [48,49], are also frequently used in therapeutic antibody development. For conventional antibodies, a short half-life might be a disadvantage; however, for antibodies targeting the receptor, a short half-life helps sdAbs avoid long-term action in the body, which could affect the normal physiological functions of the receptor.

In this study, we obtained a series of anti-TfR1 VHH antibodies, among which 18N18 exhibited a good neutralizing effect against two types of JUNV pseudoviruses compared with other candidate antibodies. Its IC_50_ against the HIV-vectored pseudovirus was 1.1168 nM. Moreover, in neutralization against the VSV-vectored pseudovirus, 18N18 also showed better neutralization, with an IC_50_ of 0.0136 μM, compared with 18N20-1 and 18N20-2. Notably, it was found that the binding activity of the antibodies to the TfR1 apical domain (Figure 3D) was not consistent with their neutralization activity **(**Figure 4C). For instance, both 18N13-1 and 18N18 exhibited strong binding to TfR1 apical domains, but 18N13-1 did not exhibit high neutralizing activity, which might be because 18N13-1 did not bind to the neutralizing epitopes. We also analyzed the binding epitopes of these antibodies to TfR1, including the detection of the antibodies’ binding activity to the TfR1 apical domain by ELISA, as well as the docking of 18N18 with TfR1 using AlphaFold 3. As an antibody targeting the human receptor, 18N18 exhibited a cross-protective ability against pseudotype of another New World arenavirus, MACV. Analysis of the interaction between 18N18 and hTfR1 confirmed that 18N18 blocked the binding of JUNV to hTfR1. In the future, the broad-spectrum neutralization ability of 18N18 could be investigated.

## Figures and Tables

**Table 1:** The binding ability of the candidate antibodies.

	ELISA		SPR		
	EC_50_ (μg/mL)	EC_50_ (pM)	Ka (1/Ms)	Kd (1/s)	KD (M)
18N13-1	0.00375	41.6667	3.44 × 10^5^	2.26 × 10^−4^	6.56 × 10^−10^
18N13-2	0.003874	43.0444	3.90 × 10^6^	3.21 × 10^−4^	8.23 × 10^−11^
18N14-1	0.004792	53.2444	5.64 × 10^5^	1.71 × 10^−4^	3.03 × 10^−10^
18N14-2	0.005941	66.0111	5.06 × 10^5^	1.63 × 10^−4^	3.22 × 10^−10^
18N14-3	0.005106	56.7333	1.47 × 10^6^	2.89 × 10^−4^	1.96 × 10^−10^
18N14-4	0.007071	78.5667	5.83 × 10^5^	4.18 × 10^−4^	7.17 × 10^−10^
18N14-5	0.003991	44.3444	5.36 × 10^6^	1.08 × 10^−4^	2.01 × 10^−11^
18N15	0.005134	57.0444	2.85 × 10^5^	6.69 × 10^−5^	2.35 × 10^−10^
18N17	0.009917	110.1889			
18N18	0.004461	49.5667	2.71 × 10^6^	3.25 × 10^−5^	1.20 × 10^−11^
18N20-1	0.004589	50.9889	1.50 × 10^7^	2.73 × 10^−4^	1.81 × 10^−11^
18N20-2	0.004359	48.4333	8.49 × 10^6^	7.79 × 10^−5^	9.18 × 10^−12^

SPR—surface plasmon resonance; EC_50_—50% effective concentration of the antibodies; Ka—association rate constant; Kd—dissociation constant; KD—affinity constant.

## Data Availability

Data is contained within the article or Supplementary Materials.

## Supplementary Materials

The following supporting information can be downloaded at: https://www.mdpi.com/article/10.3390/v16121951/s1: Figure S1: Construction of the Anti-TfR1 Nanobody Library; Figure S2: SDS-PAGE analysis of candidates; Figure S3: Affinity of candidates to hTfR1 (measured using SPR); Figure S4: Binding ability of ch128.1 to hTfR1^D184-E383^; Figure S5: The docking model of 18N13-1/18N15 with hTfR1; Table S1: Thermostability of candidates.
