# Supplementary material for: A hTfR1 Receptor-Specific VHH Antibody Neutralizes Pseudoviruses Expressing Glycoproteins from Junín and Machupo Viruses
Source: Viruses. 2024 Dec 20;16(12):1951. doi: 10.3390/v16121951 (PMC11680233; doi:10.3390/v16121951)
Supplement: Supplementary file 1 [file viruses-16-01951-s001.zip › viruses-3301314-supplementary.pdf]

Figure S1

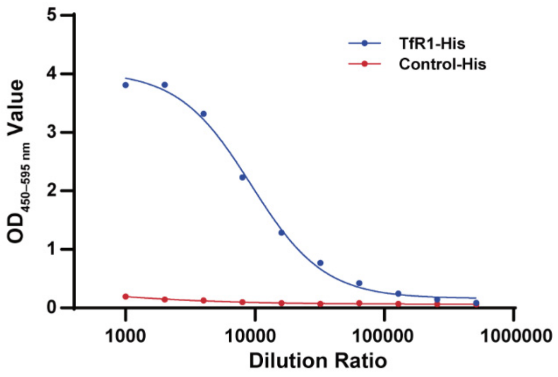

Figure S1. Detection of the IgG specificity to TfR1 in serum.
Detection of anti-hTfR1 immunoglobulin G (IgG) titer in alpaca serum was performed using an enzyme-linked immunosorbent assay (ELISA). A horseradish peroxidase (HRP)-conjugated goat anti-Llama IgG H&L antibody, was used for the detection of specific IgG. An irrelevant antigen with His-tag was set as a negative control.

Figure S2

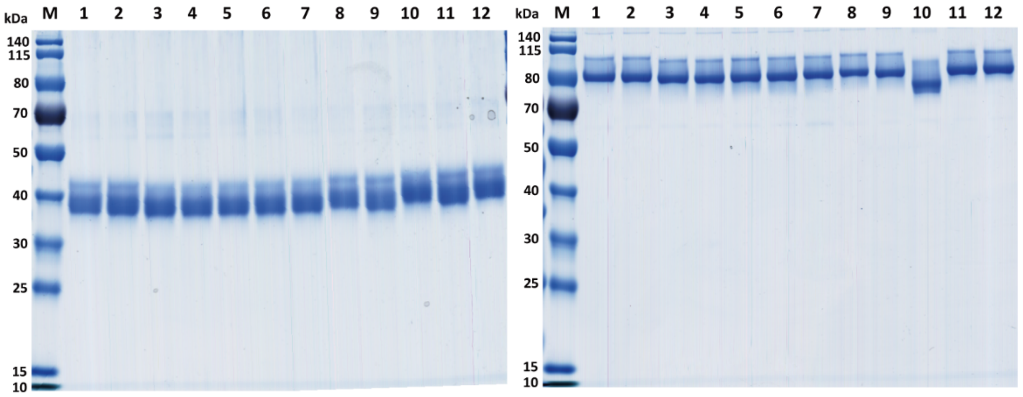

Figure S2. SDS-PAGE analysis of candidates.

SDS-PAGE analysis of 12 candidate VHH antibodies under non-reducing (right) and reducing (left) conditions: M: marker, Lane 1: 18N13-1, Lane 2: 18N13-2, Lane 3: 18N14-1, Lane 4: 18N14-2, Lane 5: 18N14-3, Lane 6: 18N14-4, Lane 7: 18N14-5, Lane 8: 18N15, Lane 9: 17, Lane 10: 18N18, Lane 11: 18N20-1, Lane 12: 18N20-2.

Figure S3

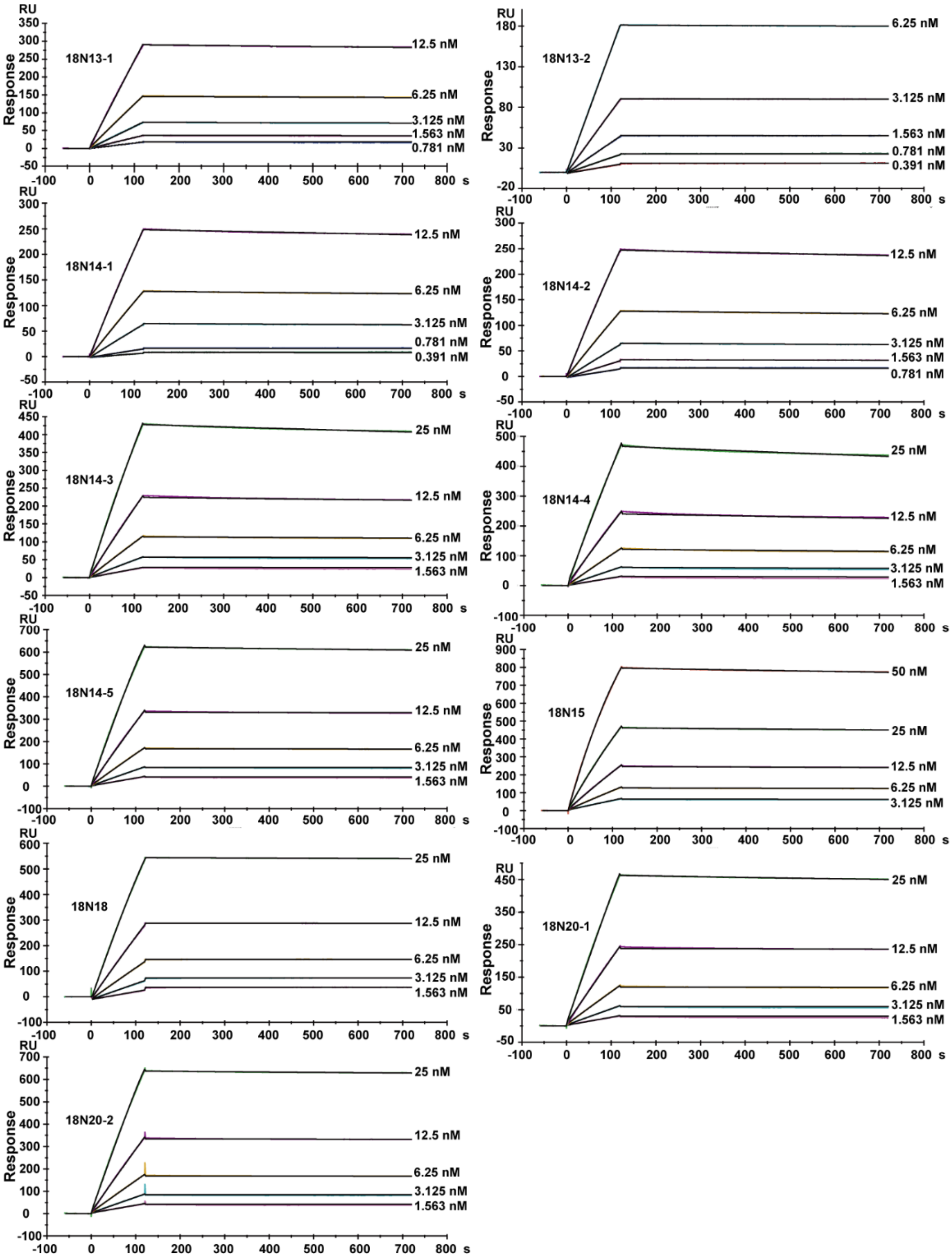

Figure S3. Affinity of candidates toward hTfR1 (detected using SPR).

Affinity detection of the candidates binding to hTfR1 using surface plasmon resonance (SPR). The hTfR1 was diluted in five successive gradients. The data was processed using Biacore T200 Evaluation Software, according to a 1:1 binding model.

Figure S4

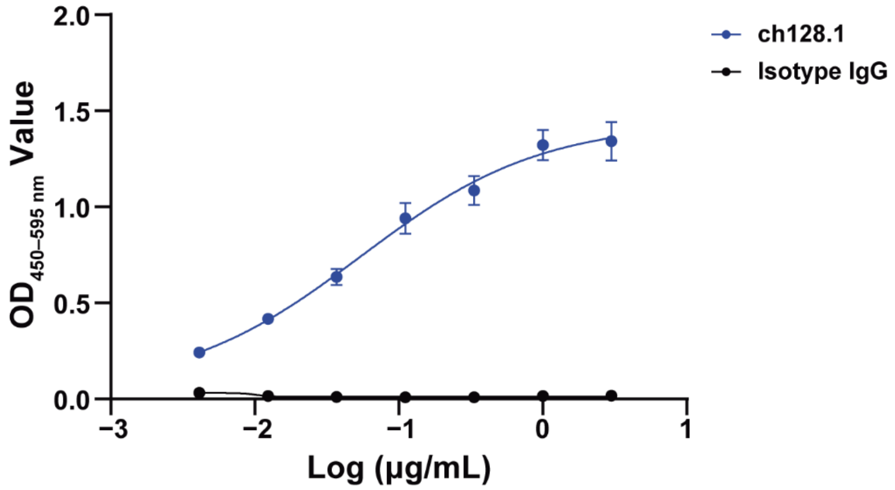

Figure S4. Binding ability of ch128.1 to hTfR1$^{D184\text{-}E383}$.

Detection of binding ability of ch128.1 with hTfR1$^{D184\text{-}E383}$ by ELISA. The secondary antibody was an HRP-conjugated goat anti-human IgG antibody. An isotype IgG was set as a negative control.

Figure S5

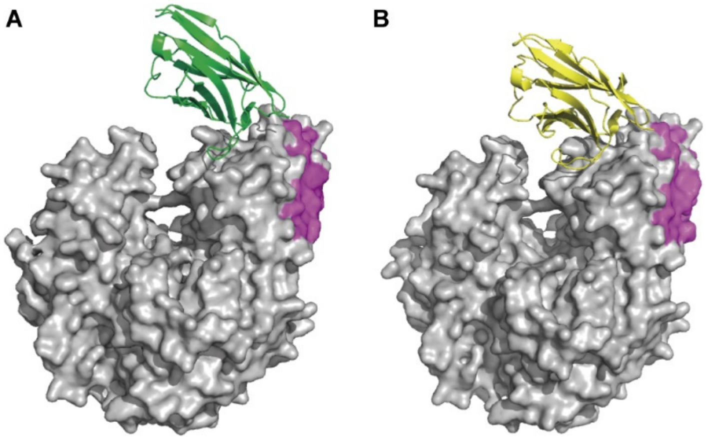

Figure S5. The docking model of 18N13-1/18N15 with hTfR1.

The binding of 18N13-1 (A) or 18N15 (B) to hTfR1 was performed by AlphaFold 3. The binding epitope of JUNV GP1 to TfR1 was labeled in pink. The structures of 18N13-1 and 18N15 were shown in green and yellow, respectively.

Table S1 Thermostability of the candidate antibodies

Sample	Tm1		Tm2		Tm3		Tagg 266	
	Tm1 (°C)	Average (°C)	Tm2 (°C)	Average (°C)	Tm3 (°C)	Average (°C)	Tagg 473 (°C)	Average (°C)
18N13-1	73.5	73.05	80.9	80.62	-	-	69.6	69.45
	72.6		80.33		-		69.3	
18N13-2	65.6	65.65	75.1	75.25	-	-	74.12	74.01
	65.7		75.4		-		73.89	
18N14-1	66.12	66.05	73.2	73.2	-	-	67.29	67.1
	65.98		73.2		-		66.9	
18N14-2	66.02	65.97	-	-	-	-	70.2	70
	65.91		-		-		69.8	
18N14-3	66.2	66.49	-	-	-	-	67.72	67.86
	66.78		-		-		68	
18N14-4	65.75	65.8	73	72.85	-	-	73.51	73.65
	65.84		72.7		-		73.78	
18N14-5	67.1	67.14	76.1	76.18	-	-	75.13	74.88
	67.17		76.25		-		74.63	
18N15	62.41	62.75	70.61	70.86	79.29	78.86	64.64	64.47
	63.08		71.11		78.43		64.3	
18N18	64.39	64.42	78.37	78.3	-	-	78.3	78.25
	64.45		78.22		-		78.2	
18N20-1	66.09	66.09	77.59	77.55	-	-	69.48	69.68
	66.08		77.5		-		69.88	
18N20-2	67.03	67.04	80.5	80.43	-	-	74.39	74.41
	67.04		80.36		-		74.42	
